# Strategies for Isolating and Propagating Circulating Tumor Cells in Men with Metastatic Prostate Cancer

**DOI:** 10.3390/diagnostics12020497

**Published:** 2022-02-15

**Authors:** Gerit Theil, Joanna Bialek, Christine Weiß, Felix Lindner, Paolo Fornara

**Affiliations:** Medical Faculty of Martin Luther University Halle-Wittenberg, University Clinic and Outpatient Clinic for Urology, 06120 Halle (Saale), Germany; joanna.bialek@uk-halle.de (J.B.); tine.weiss@gmx.de (C.W.); felix.lindner@uk-halle.de (F.L.); paolo.fornara@uk-halle.de (P.F.)

**Keywords:** circulating tumor cells, prostate cancer, isolation platforms

## Abstract

Selecting a well-suited method for isolating/characterizing circulating tumor cells (CTCs) is challenging. Evaluating sensitive and specific markers for prostate cancer (PCa)-specific CTC identification and analysis is crucial. We used the CellCollector EpCAM-functionalized system (CC-EpCAM) and evaluated and developed a PCa-functionalized version (CC-PCa); we then compared CTC isolation techniques that exploit the physical and biological properties of CTCs. We established two cohorts of metastatic PCa patients (mPCa; 15 in cohort 1 and 10 in cohort 2). CTC cultivation experiments were conducted with two capturing methods (Ficoll and ScreenCell). The most sensitive detection rates and highest CTC counts were reached with the CC-PCa and ScreenCell system. Patients with ≥5 CTCs isolated with CC-EpCAM had an overall survival (OS) of 0.93 years, and patients with ≥5 CTCs isolated with CC-PCa had an OS of 1.5 years in cohort 1. Nevertheless, we observed the highest sensitivity and specificity for 24-month survival by the Ficoll with CD45 depletion and ScreenCell system with May-Grunwald Giemsa (MGG) staining. The EpCAM molecule is an essential factor related to OS for CTC isolation based on biological properties in mPCa patients. The best-suited CTC capture system is not limited to one characteristic of cells but adapted to downstream analysis.

## 1. Introduction

Circulating tumor cells (CTCs) have been one of the most discussed biomarkers for monitoring cancer progression in the last decade. They are individual tumor cells or cohorts of cells from primary tumors or metastases that may invade the vasculature in adjacent tissue [1,2]. In the blood of cancer patients, CTCs might reflect important information on current tumor progression and cancer treatment. Furthermore, CTCs represent a heterogeneous population, and only a very small percentage (<<0.01%) of the millions of cells that enter the blood system daily have the ability to form metastases. During dissemination, multiple steps and mechanisms are involved in phenotypic changes in CTCs.

Epithelial–mesenchymal transition (EMT) is a significant mechanism by which CTCs invade the surrounding stroma and blood circulation [3]. In this process, CTCs lose epithelial markers, such as epithelial cell adhesion molecules (EpCAM) and E-cadherin, and show a mesenchymal phenotype. This morphological change is generally reversible via epithelial–mesenchymal transition (MET), which includes the activation of mixed epithelial/mesenchymal CTC hybrid phenotypes [1,4]. These CTC phenotypes are also present in the blood of patients with prostate cancer (PCa) [5].

PCa is the second most diagnosed cancer, with an estimated 1.4 million new cases worldwide in 2020, and it was the fifth leading cause of death in that same year [6]. In the clinic, PCa can be present in a localized indolent or metastatic lethal stage. Individual patients demonstrate phenotypic and genomic intratumoral heterogeneity, which is challenging for diagnosis and treatment [7,8]. A single tissue biopsy sample may not reflect the current stage of the cancer. Therefore, liquid biopsy as a method that captures heterogeneous tumor material is needed. The isolation and characterization of CTCs provide such opportunity [9,10]. CTC enrichment can be achieved based on physical (size, deformability, density or electrical charge) or biological (cell-surface protein) properties [11]. Unfortunately, the gold standard CellSearch system (Silicon Biosystem, Menarini, Florence, Italy) isolates CTCs with EpCAM-coated ferrofluid beads and identifies an overexpression pattern of cytokeratin (CK) 8, 18 and 19 and the absence of CD45 expression [12,13]. This is the only Food and Drug Administration (FDA)-approved CTC isolation platform for monitoring patients with metastatic prostate (mPCa), breast and colorectal cancers [13,14,15]. However, EpCAM-based enrichment of CTCs alone does not always correspond to heterogeneity in the CTC phenotype. Furthermore, for PCa, it is crucial to evaluate sensitive and specific markers for cancer-specific CTC identification and analysis.

Prostate-specific membrane antigen (PSMA), a type II transmembrane glycoprotein, has become a clinically validated therapeutic target [16]. PSMA expression in PCa tissue is 100 to 1000 times higher than that in benign tissue [17]. Interestingly, antiandrogen treatment upregulates PSMA expression in patients with castration-resistant prostate cancer (CRPC) [18]. Low levels of PSMA can be expressed in lung cancer, colorectal carcinoma and glioblastoma [19].

Another possible marker for CTC isolation/characterization is prostate stem cell antigen (PSCA), a glycosylphosphatidylinositol (GPI)-linked cell-surface protein that is expressed in >80% of PCa patients [20]. This cell-surface protein shares 30% homology with stem cell antigen type 2 (SCA-2), a surface marker of immature lymphocytes [21]. Furthermore, PSCA expression increases with a higher Gleason grade and progression to androgen independence [22]. PSCA can also be detected in the kidney, urothelium and lung, which has been reviewed by van der Toom et al. [23] 2019.

The most commonly used marker in PCa screening, monitoring and disease progression is prostate-specific antigen (PSA, also known as kallikrein-related peptidase 3). The expression of PSA is specific for PCa, but it also tends to decrease with cancer progression and phenotypic change [7,24]. Moreover, PSA is an androgen-regulated protease, and PCa cell proliferation is highly dependent upon androgen receptor (AR) signaling [25,26]. Androgen deprivation therapy (ADT) results in a decrease in PSA secretion under the detection limit in serum. A rising PSA level is associated with the development of CRPC and the risk of metastasis [27].

Nevertheless, PSMA, PSCA and PSA are potential markers for specific enrichment of CTCs in the blood of patients with metastasized PCa. The aim of our preliminary investigation was to evaluate and develop a PCa-specific CTC isolation method. For this purpose, we used the CellCollector (CC-EpCAM), a medical wire that enables in vivo CTC isolation with a monoclonal antibody directed to the cell-surface-expressed EpCAM of CTCs in the peripheral blood [28,29,30,31]. In our trial, we functionalized the new form of the CellCollector with EpCAM antibody or with previously mentioned PCa markers (CC-PCa). In a proof of concept, we analyzed the different CellCollector system ex vivo.

We compared isolation techniques that use the physical (size and the density of cells) and biological (expression of EpCAM, PSMA, PSCA and PSA) properties of CTCs. Additionally, we aimed to determine a suitable CTC isolation method for possible CTC cultivation in advanced PCa patients.

## 2. Materials and Methods

### 2.1. Study Collective

**Figure 1:**
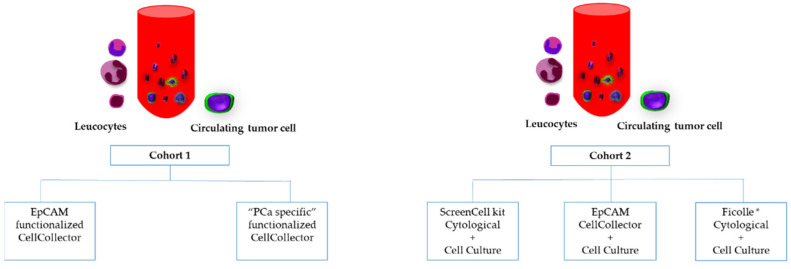
Overview of CTC isolation approaches in the two independent patient cohorts. Abbreviations: Ficoll *: Ficoll + CD45 depletion.

This prospective study was planned for the University Clinic and Outpatient Clinic for Urology, Medical Faculty of Martin Luther University Halle-Wittenberg. All patients provided written informed consent before blood collection. The protocol was approved by the medical faculty ethics committee of Martin Luther University Halle-Wittenberg (2012-65). Men were enrolled with histologically confirmed and metastatic prostate adenocarcinoma. We evaluated differential CTC capture methods in mPCa patients. For this purpose, two cohorts of PCa patients under treatment were prospectively analyzed. Blood samples from cohort 1 (*n* = 15) were analyzed with two different functionalized and spiralized CellCollector systems (GILUPI GmbH, Potsdam, Germany). In cohort 2 (*n* = 10), the ScreenCell (SC) kit (ScreenCell SA, Sarcelles, France) for cytological analysis and the SC kit for cell culture and buffy coat analysis accompanied by CD45 depletion were evaluated. The viability of CTCs was checked in cell culture approaches only in cohort 2 (Figure 1). All blood samples (EDTA tubes) were processed within 3 h.

### 2.2. Antibody Validation for the PCa-Specific Functionalization of the CellCollector System

The PCa cell line LNCaP was purchased from ATCC (www.atcc.org, accessed on 1 April 2018) and routinely maintained in RPMI 1640. The media were supplemented with 10% fetal bovine serum. The cell line was grown on sterile glass slides until it reached a confluence of 70%. The cells were fixed with ROTI-Histofix 4% (Carl Roth, Karlsruhe, Germany) for 15 min, washed with phosphate-buffered saline (PBS) (Sigma/Merck, Darmstadt, Germany), and blocked with 5% milk (Th. Geyer, Berlin, Germany) in PBS. Incubation with anti-PSMA (Cell Signaling Technology, Frankfurt, Germany), anti-PSCA (Abcam, Cambridge, UK) or anti-PSA (Cell Signaling) was performed overnight at 4 °C. The secondary antimouse antibody (DIANOVA GmbH, Hamburg, Germany) was applied the next day for 1 h at RT. Cell nuclei were visualized using Hoechst 33258 (Merck, Darmstadt, Germany). Images (60×) were taken using an inverted fluorescence microscope (Carl Zeiss Microscopy, Jena, Germany).

### 2.3. CTC Isolation Approaches Based on Biological Properties

CellCollector (CC) system was used to enrich CTC based on expression of the FDA-approved surface marker EpCAM (CC-EpCAM) [13]. Furthermore, we developed a PCa-specific functionalization of the CellCollector system (CC-PCa). This was performed with a combination of 4 different antibodies against PSMA, PSA, PSCA and EpCAM. The concentration of the antibodies was 7.5 µg/mL; in summary, 20 µg antibodies were coupled on the spiral tip.

The single steps of the procedure were performed as described in Theil et al. [32]. We used 16 cm-long spiraled medical stainless steel wires. The 4 cm spiraled tips of the wire were previously covered with a thick (0.2 µm) layer of gold and a polycarboxylate layer (1–5 µm). We incubated them for 15 min in sterile distilled water to rehydrate the hydrogel and then activated them in 1-ethyl-3-(3-dimethylaminopropyl) carbodiimide hydrochlo-ride/*N*-hydroxysulfosuccinimide (EDC/NHS) solution (Sigma) for 20 min at 22 °C. Next, a 100 mM solution of NHS in 50 mM 2-(*N*-morpholino)ethane-sulfonic acid (MES) buffer (Sigma), 0.5% EDC (Sigma) was added. Finally, the wire was rinsed using 5 mM acetic acid (Roth) and incubated with EpCAM or PSCA, PSMA, PSA and EpCAM antibodies for 1 h at 22 °C to achieve covalent bonding between the hydrogel and the antibody. To block the free carboxyl groups, the hydrogel-covered wire was incubated with 1 M ethanolamine hydrochloride (Sigma) at pH 8.5. After washing with distilled water, the wires were stored at 4 °C until use.

In the following step, the performance of CC-EpCAM and CC-PCa was demonstrated in spiking experiments. Single LNCaP cells were spiked into healthy donor blood, and CellCollector wires were placed into the blood in a 7.5 mL EDTA tube, positioned on a rotating platform at room temperature and incubated for 60 min. At the end of the incubation, the wires were immediately placed in PBS and rinsed three times in a tube with new PBS solution. The cells were fixed on the wire with a 10 min acetone treatment and blocked with 3% bovine serum albumin/PBS for 30 min. Patient blood samples were subjected to the same conditions. By in vivo application, the wire would be inserted into the cubital vein through a 20G cannula and remain in place for 30 min. The CC-PCa was not approved for in vivo; for this reason, we compared the different wires ex vivo.

### 2.4. CTC Isolation Approaches Based on Physical Properties

The ScreenCell Cyto kit (ScreenCell SA, Sarcelles, France) isolates CTCs based on their size (<8 µm) with a filter system. All cells smaller than 8 µm are run through the filter. We performed the analysis using 3 mL of blood according to the manufacturer’s instructions (ScreenCell, Sarcelles, France) for cytomorphology characterization of the remaining cells.

Three milliliters of EDTA blood was diluted in 4 mL filtration buffer and incubated for 8 min at room temperature. After incubation, 7 mL of diluted blood was filtered, and the filter device was washed with PBS. The filter was released onto absorbing paper and dried at room temperature for 15 min. After drying, cytomorphology characterization was performed.

The second CTC isolation method was based on the density of the cells and the CD45 depletion step using preconjugated, anti-CD45 magnetic beads (Dynabeads CD45, Life Technologies, Carlsbad, CA, USA). CTC enrichment was succeeded by density gradient separation with Histopaque-1077 (Sigma-Aldrich, Steinheim, Germany). Three milliliters of Histopaque-1077 was transferred to a sterile 15 mL tube and carefully overlayered with 3 mL of blood. This sample was centrifuged for 30 min at 500 g without braking to separate mononuclear cells (including CTCs) located at the interface between the plasma (upper layer) and the Ficoll-Histopaque (bottom). The interface was gently removed to avoid disturbing the layering, transferred to a new sterile tube and washed 3 times with sterile PBS. Recovered cells were resuspended in 500 µL PBS and prepared for microscopical examination (cytospins) by a cytocentrifuge (Epredia Cytospin 4 Zytozentrifuge, ThermoScientific, Waltham MA, United States) at 1000 rpm for 4 min at room temperature. The cytospins (ten slides) dried at room temperature (RT) for a minimum of 30 min and identified by cytomorphological or fluorescent immunohistochemistry characterization.

### 2.5. Ex Vivo Culture of CTCs

For cultivation with the ScreenCell Kit (ScreenCell Cell Culture Kit, ScreenCell SA, Sarcelles, France), we used 7 mL of diluted blood (6 mL of blood to 1 mL of ScreenCell LC dilution buffer). After incubation for 2 min, 1.6 mL of culture medium was added and homogenized once by inverting the tube, and filtration was started. Subsequently, the filter was released into a tissue culture plate filled with 600 µL culture medium.

CTCs from 3 mL of blood with a Ficoll * gradient were prepared for cultivation, as mentioned above, by adding 500 µL of cultivation medium.

The medium consisted of RPMI Medium 1640 (Life Technologies, Carlsbad, CA, USA), recombinant human epidermal growth factor 20 ng/mL (Life Technologies), recombinant human basic fibroblast growth factor 50 ng/mL (Life Technologies), 1% penicillin–streptomycin mix (Life Technologies) and 1 mL/50 mL B27 supplements, minus vitamin A (Life Technologies). No androgens and glucocorticoids were additionally included.

After gentle mixing, 30 µL of the cell suspension was transferred into wells of a 12-well cultivation plate. Furthermore, 20 µL of cell suspension was added to a well of a 10-well CELLview slide (Greiner Bio-One, Frickenhausen, Germany). CTCs were cultivated under standard culture conditions (37 °C and 5% atmospheric CO_2_) and observed by inverted microscopy. We changed a third of the medium after the 10th, 13th and 22nd days of cultivation and determined the PSA level in the samples.

### 2.6. Staining an Enumeration of Collected Cells

The cells isolated by the SC filter and Ficoll * were stained with May-Grunwald Giemsa staining. Staining was performed according to the manufacturer’s instructions. The filter was incubated for 2 min in May-Grunwald solution and for the next 10 min in Giemsa solution. After that, the filters and slides were washed with distilled water and dried for 15 min at room temperature.

The captured cells were fixed and blocked on the wire surface or on the Ficoll ** slides. The cells were identified as CTCs by immunofluorescence staining using pan-CK- and Hoechst-33258-positive and CD45-negative criteria. The CTCs met the following cytology-based FDA definition: (i) size ≥4 µm, (ii) visible cytoplasm, (iii) high nuclear/cytoplasm ratio, (iv) positive fluorescence staining of CK 8, 18 and 19 with negative staining of CD45 and (v) 50% of the nucleus contained within the CK border enumeration [33].

### 2.7. PSA Measurement

The PSA secretion of the cultivated CTCs in the supernatant was determined with an IMMULITE 1000 Immunoassay System (SIEMENS Healthineers, Erlangen, Germany). We used the third-generation PSA assay, which provides a very low PSA detection limit of 0.005 ng/mL.

### 2.8. Statistical Analysis

Since the sample size was rather small, we did not perform an extensive statistical analysis. We applied the Mann–Whitney test to compare continuous clinical and demographic parameters in our cohorts. Additionally, several other nonparametric tests were performed as specified in the Results section. Finally, for identification of possible correlations between the different methods, Spearman’s rank correlation coefficient (r_s_) was determined. The reported *p* values were two-sided, and ≤0.05 was considered significant. The accuracy of the CTC isolation methods was evaluated by receiver operating characteristic (ROC) analysis. Kaplan–Meier analysis was used to analyze overall survival (OS) depending on CTC count [34,35]. All statistical analyses were performed using GraphPad Prism software version 9.

## 3. Results

### 3.1. Patient Characteristics and Treatments

All the patients in our two independent cohorts had histologically and radiologically confirmed mPCa. Patients with a second cancer diagnosis were not included in this analysis. We enrolled the patients randomly in a timeline of 6 months for every cohort. Genetic characterization of primary tumor or metastasis were not performed. The clinical and pathological parameters of the patients are summarized in Table 1. A statistically significant difference was observed for only one of the surgical procedures (transurethral resection of the prostate) in the cohorts. The median age of cohort 1 was 74 years and that of cohort 2 was 63.5 years (*p* = 0.03). Twelve patients (80%) in cohort 1 and nine patients (90%) in cohort 2 had a Gleason score of more than 7 at the time of diagnosis. At the time of CTC sample collection, all patients received ADT and/or chemotherapy. The median PSA level was 13 ng/mL (0.02–353 ng/mL) in cohort 1 and 71.9 (1.7–184 ng/mL) in cohort 2 (*p* = 0.8). More than 86% of patients in cohort 1 and 100% in cohort 2 had bone metastasis. Lymph node metastasis was present in 41.7% of cohort 1 and in 50% of the patients in cohort 2. One patient in cohort 2 was only under ADT and presented multiple bone and lymph node metastases.

### 3.2. Identification of Suitable Antibodies for PCa-Specific Functionalization of the CellCollector System

**Figure 2:**
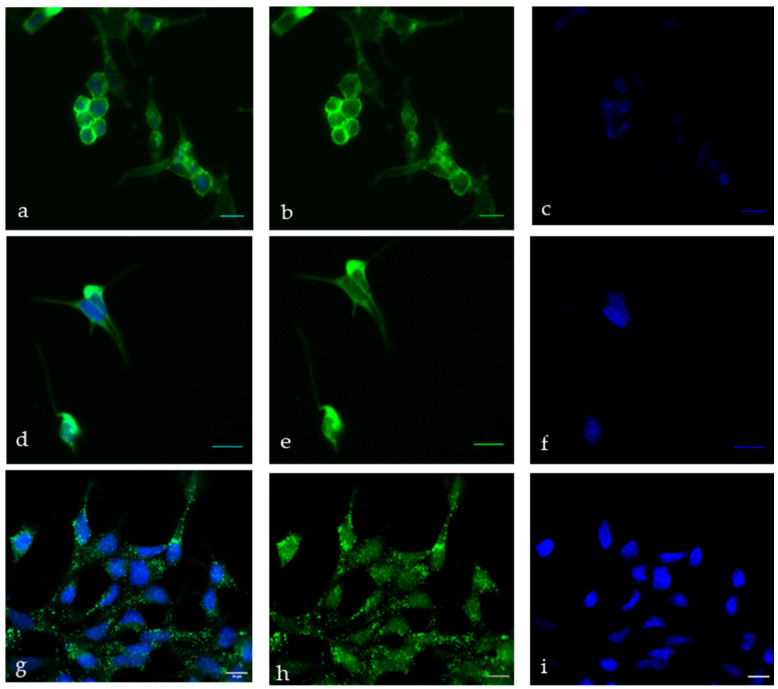
Immunofluorescence staining of LNCaP cells for (**b**) PSMA, (**e**) PSA, (**h**) PSCA and (**c**,**f**,**i**) Hoechst 33258 was used for nuclear counterstaining. Overlay of nuclear staining and marker-specific staining showing (**a**) PSMA, (**d**) PSA and (**g**) PSCA. Scale bares indicate 20 µm.

First, to implement CC-PCa for CTC capture, we checked and identified suitable antibodies compatible with the functionalization procedure and effective CTC capture. Immunofluorescence analysis revealed precise patterns of the antibodies used in LNCaP cells. The PSMA (12702S) antibody was used to detect the extracellular domain of the type II transmembrane protein. We confirmed this signal by immunostaining LNCaP cells (Figure 2b). The PSA signal of the antibody (5877S) displayed a strong cytoplasmic portion in the LNCaP cells (Figure 2e). The recombinant amino acid fragment corresponding to amino acid 1 to the C-terminus of human PSCA was used to generate the polyclonal PSCA antibody (ab 220101). We observed predominantly cytoplasmic and marginal cell membrane signals of the PSCA antibody (Figure 2h).

To check the functionality of the prepared CellCollector wires, we spiked LNCaP cells (200 cells) into healthy donor blood and evaluated the detection rate of the CC-PCa and CC-EpCAM systems. These samples (*n* = 3) were analyzed on one batch. Recovery rates from 48% for CC-PCa (PSMA, PSCA, PSA and EpCAM) and from 30% for CC-EpCAM were observed.

### 3.3. Comparison of CTC Detection Methods

**Figure 3:**
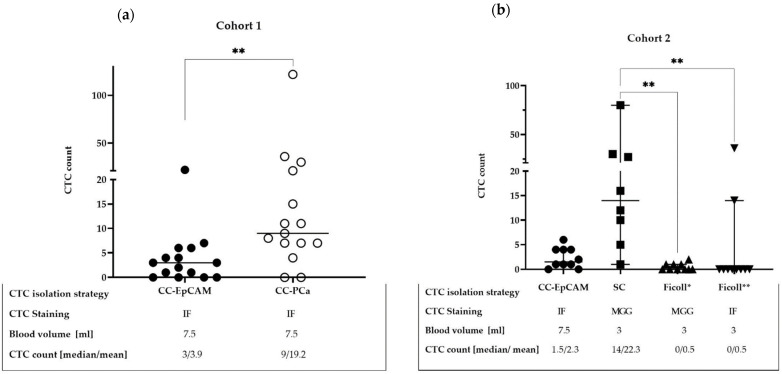
Direct comparison of CTC detection: (**a**) with the CC-EpCAM and CC-PCa systems, and the Wilcoxon matched-pairs signed-rank test was used, *p* ** 0.002. (**b**) CC-EpCAM, ScreenCell Cyto device (SC) and Ficoll gradient with CD45 depletion Ficoll *-May-Grunwald Giemsa (Ficoll *-MGG) and Ficoll **-immunofluorescence staining (Ficoll **-IF) systems and Dunn’s multiple comparison test were used. Ficoll **-IF vs. SC-MGG, *p* = ** 0.002 and Ficoll *-MGG vs. SC, *p* = ** 0.002.

**Figure 4:**
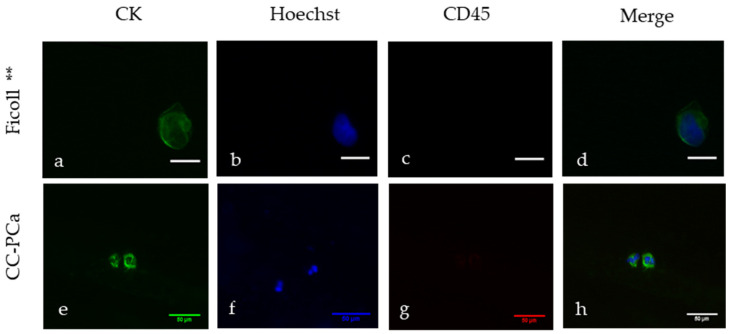
Representative CTC images (**a**,**e**) and negative leukocyte staining (**c**,**g**) from selected patients of our cohorts collected by Ficoll ** (**a**–**d**) and CC-PCa (**e**–**h**). CTCs were defined as cells with positive staining for CK (**a**,**e**) but negative staining for CD45 (**c**,**g**). Hoechst staining showed the presence of cell nucleoli (**b**,**f**). The scale bars indicate 20 µm, and cells were scanned at a magnification of 63.3× (**a**–**d**). The scale bars indicate 50 µm, and the cells were scanned at a magnification of 20× (**e**–**h**). Merge images are presented in (**d**,**h**).

Different CTC isolation methods were analyzed in the two independent cohorts of mPCa patients (Figure 3). Positive CTC patients in our cohorts were defined as having ≥1 CTC in the blood sample. In cohort 1, we compared the different functionalized CellCollector systems with spiralizer tips (Figure 3a). With CC-PCa, we detected ≥1 CTC in 12 out of 15 (86.7%) patients (Figure 4e–h); with CC-EpCAM, we detected ≥1 CTC in 11 out of 15 (73.3%) patients. The median CTC count detected with CC-PCa was nine (range 0–122) and that with CC-EpCAM was three (range 0–22). Additionally, the Wilcoxon matched-pair analysis demonstrated a median of four CTC differences when comparing CC-PCa to CC-EpCAM (*p* = 0.002). Interestingly, only a weak correlation was observed between the different functionalized wires (Spearman rank correlation coefficient r_s_ = 0.37, *p* = 0.17).

**Figure 5:**
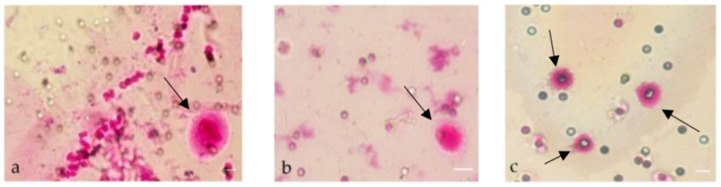
CTCs stained with MGG present in blood samples of PCa patients: (**a**,**b**) Patient #7 current treatment enzalutamide after docetaxel chemotherapy with a PSA level of 105 ng/mL. (**c**) Patient #5 current treatment docetaxel chemotherapy with a PSA level of 5.2 ng/mL. Scale bares indicate 20 µm. Arrows indicate CTCs.

In cohort 2, three different CTC isolation techniques based on the physical and biological CTC characteristics were compared, and two different staining methods for identification were used. The CTC detection rates based on the density (Ficoll *) and CD45 depletion were 2 out of 10 (20%) with immunofluorescence staining (Ficoll **) (Figure 4a–d) and 4 out of 10 (40%) with MGG staining (Ficoll *). The SC filter with MGG staining based on the size of the cells was used to detect CTCs in 8 out of 10 (80%) patient samples (Figure 5). Two of the ten SC probes could not be evaluated because of blood clots on the filters.

CC-EpCAM detected CTCs per 7 mL in 8 out of 10 (80%) cohort 2 patients (Figure 3b). To better compare all the CTC detection rates (≥1 CTC), we calculated the blood volume needed for CC-PCa to be 3 mL and detected CTCs in 5 out of 10 (50%) blood samples. The median CTC count was 0.65 CTC (range 0–6 CTCs) per 3 mL of blood for CC-EpCAM and 0 CTCs for Ficoll * (range 0–2) and Ficoll ** (range 0–36 CTCs). The SC filter captured a median of 14 CTCs per 3 mL of blood (range 1–79 CTCs) (Figure 3b). The CTC count of the Ficoll * filter correlated moderately with the SC filter, r_s_ of 0.54, but was not significant (*p* = 0.18). In addition, the CTC count for CC-EpCAM with Ficoll * correlated with a moderate r_s_ of 0.47 (*p* = 0.16). Significant differences in CTC count were demonstrated between CS-MGG vs. Ficoll * (*p* = 0.002) and CS-MGG vs. Ficoll ** (*p* = 0.002). For the analysis, Dunn’s multiple comparison test was used (Figure 3b).

The PSA level demonstrated no significant correlation with the CTC isolation method in either cohort.

To evaluate the sensitivity and specificity of the different isolation methods for predicting survival, we determined the receiver operating characteristic (ROC) and area under the curve (AUC) of the survival models. The CTC cutoff was ≥1 CTC. The results demonstrated that for a survival time of 24 months, the AUC values of the CTC isolation methods ranged from 0.53 to 0.79 (Table 2). Interestingly, the efficacy of CTC isolation with Ficoll * (AUC = 0.79) was higher than that of CC-EpCAM and CC-PCa in cohort 1 and that of CC-EpCAM, SC and Ficoll ** in cohort 2 (AUC = 0.53, 0.67, 0.55, 0.73 and 0.67, respectively, Table 2). Furthermore, the AUCs (0.73 and 0.79) of the CTC isolation methods, which used MGG staining, were the highest (SC and Ficoll *).

Additionally, the isolation methods used required equivalent professional laboratory skills of the staff. The working time per method ranged from >2 h for the SC system to >3 h for the other three systems (Table 3). The SC filter device provided rapid CTC isolation with a single wash step. The CellCollector and the Ficoll gradient systems required more than three wash steps. We observed a higher number (≥500) of contaminating leukocytes when we used the size-based and density-based CTC isolation methods. Lower leukocyte contamination by the Ficoll system was reached by the addition of a CD45 depletion step. However, the CellCollector system had the lowest leukocyte contamination. Of the four isolation methods, only the Ficoll * method was able to isolate CTCs that could be cultivated. Finally, all the systems required trained and experienced observers to identify and image the CTCs.

### 3.4. CTC Cultivation

**Figure 6:**
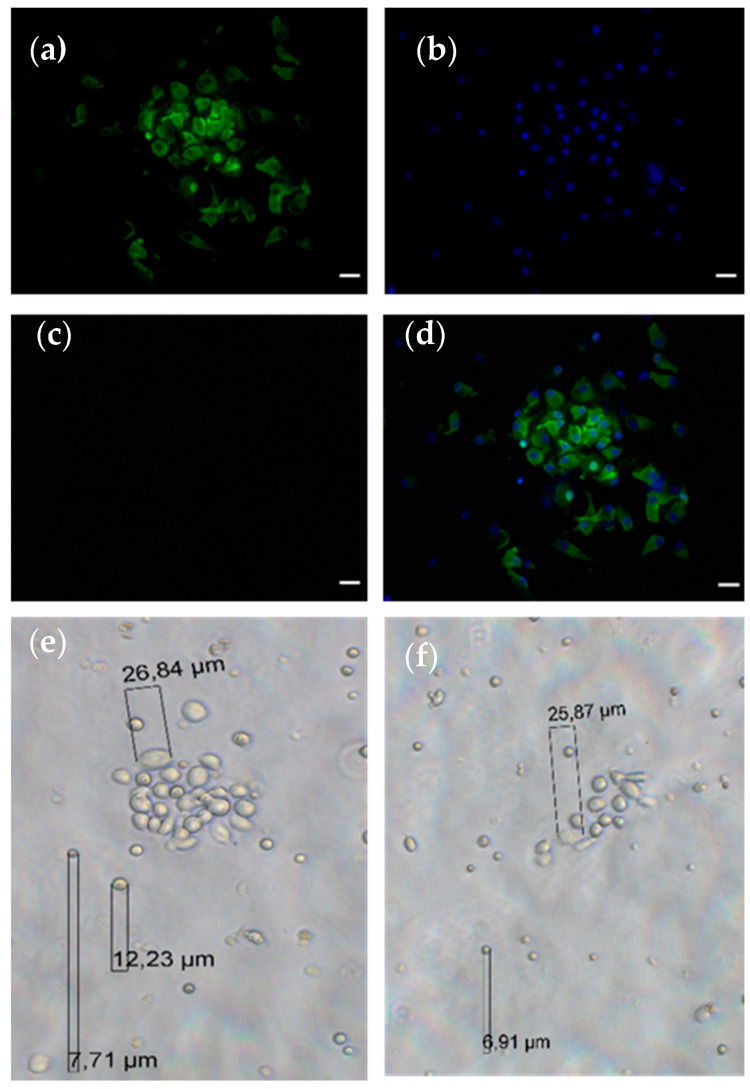
Representative images of cultivated CTCs. The CTCs display positive staining for (**a**) CK 8, 9 and 18, (**b**) Hoechst 33258 staining, (**c**) CD45 negativity and (**d**) overlay of images (**a**–**c**). Scale bar presents 20 µm. Brightfield images of growing CTCs (**e**,**f**).

Blood samples of three mPCa patients in cohort 2 were processed parallel with Ficoll * (3 mL of blood) and CS circular filter CTC cultivation (6 mL of blood). The originating CTCs were directly cultivated in a 24-well cell culture and seeded in CELLview slides. However, the cultivation of CTCs was possible only for one patient, for whom isolation with the Ficoll * system was performed. The patient had bone and lymph node metastasis of the PCa and was treated at the time of blood sampling with ADT. The serum PSA level was 10.7 ng/mL. In addition, he had not received any chemotherapy. After 7 days of culture, we changed a third of the medium, and on the 10th, 13th and 22nd days of cultivation, we determined the PSA level in the collected medium. Furthermore, CTCs were characterized for CK expression (Figure 6a). The cultivated CTCs showed positivity for classical epidermal markers (CK 8, 9 and 10). Single CTCs were also positive for PSA expression by immunostaining (data not shown). The CTCs demonstrated by brightfield imaging mainly adhered to growing cells, and clustering of the cells was observed. The morphologies of the growing CTCs became paler and more elongated in the culture (Figure 6e,f).

**Figure 7:**
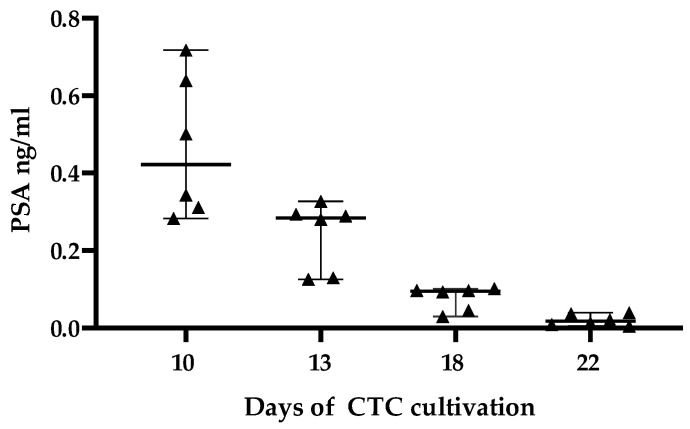
Concentration of PSA in ng/mL of cultivated CTCs in the supernatant displayed as median with range. CTCs were successfully cultivated in different single-cell culture wells of a 24-well plate. CTCs originated from a single patient.

Furthermore, we analyzed the PSA level in the cell culture wells of the cultivated CTCs. After ten days of cultivation, we determined a mean PSA level of 0.47 ng/mL in the tested wells. Unfortunately, a mean PSA level of only 0.02 ng/mL was detected in the wells over 3 weeks (Figure 7).

### 3.5. Prognostic Performance of the CTC Status

**Figure 8:**
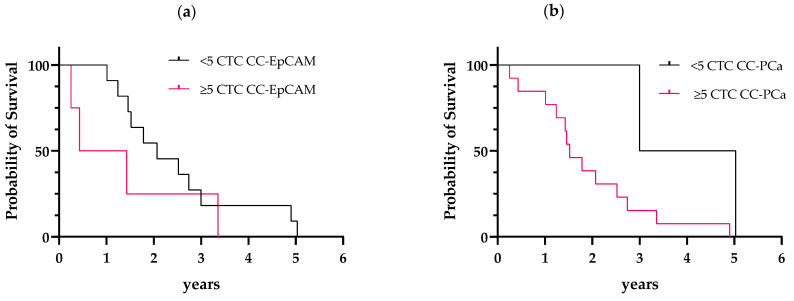
Kaplan–Meier curves of OS according to CTC count determined with (**a**) CC-EpCAM and with (**b**) CC-PCa: (**a**) The patients with <5 CTCs and ≥5 CTCs showed a difference in OS (2.1 years versus 0.93 years (HR 0.53, 95% CI: 0.13–2.1)). (**b**) The patients with <5 CTCs and ≥5 CTCs showed a difference in OS (4.0 years versus 1.5 years (HR 0.33, 95% CI: 0.11–0.97)).

Patients of our cohorts were positive for PCa metastasis, which resulted in a slight decrease in general conditions. In study cohort 1, the patients reached a follow-up time of 5 years. We used the established CTC cutoff values of <5 or ≥5 CTCs for the OS analyses [13]. The OS estimated in different groups was compared using the log-rank (Mantel–Cox) test. Patients (73.3%) evaluated with CC-EpCAM and with <5 CTCs survived a median of 2.1 years, and patients (26.7%) with ≥5 CTCs survived a median of 0.93 years. The hazard ratio (HR), referring to <5 CTCs or ≥5 CTCs, was 0.53 (95% confidence interval (CI): 0.13–2.1, Figure 8a).

Patients (20%) evaluated with CC-PCa and with <5 CTCs survived a median of 4.0 years, and patients (80%) with ≥5 CTCs survived a median of 1.5 years. The HR, referring to <5 CTCs or ≥5 CTCs, was 0.33 (95% CI: 0.11–0.97, Figure 8b). In cohort 2, we reached a follow-up time of 2 years. Unfortunately, three patients were lost to follow-up. Due to the small study population, we could not perform survival analyses.

## 4. Discussion

Selecting a well-suited method for isolating/characterizing CTCs remains a challenge. Isolation platforms should be used to detect most heterogeneous CTC phenotypes that survive after drug therapy to identify treatment targets for clinical testing or drug development [4,36,37]. In our study, we evaluated the CTC count with isolation platforms relying on different cell characteristics and laboratory handling. We assessed two cohorts of mPCa patients. The demographical characteristics were not significantly different in our study population. In addition, we used in our cohorts at least one CTC capturing method, based on the EpCAM signal currently recognized at the only FDA-approved surface marker for CTC detection. We performed immunofluorescence staining of the cytology-based FDA CTC criteria. These facts allowed for a more rigorous discussion of the results.

The first step was to develop and evaluate the PCa functionalized CellCollector system as a proof of concept. Our immunofluorescence displayed the classical signals in the analyzed LNCaP cells. The only polyclonal antibody, PSCA, was created against the whole amino acid sequence of the protein. For that reason, this antibody can bind in any position of the protein. This reflects the signals in our analyzed cells. Wang et al. demonstrated in Western blot analyses the expression of PSCA in LNCaP cells [38]. Unspecific capturing of cells in our trial was excluded by the use of the cytology-based FDA definition of CTCs. We revealed in the spiking experiment a moderate recovery rate (30–48%) of the cells with two different CellCollector systems. One reason for this could be the expression of various adhesion molecules of cell line cells that leads to different cell–cell contacts that can potentially influence the recovery rate [39]. Furthermore, in our previous trial, we received with the EpCAM functionalized CellCollector a high recovery rate (35%) of LNCaP (200 cells) and low recovery rate of 10% (50 cells) [32]. This suggested a higher sensitivity for capturing PCa cells with the CC-PCa. 

Our data for cohort 1 highlighted the disparities in the detection rate and CTC count in matched blood samples between the different CellCollector systems. These results show detection rates of 86.7% for CC-PCa vs. 73.3% for CC-EpCAM and a significantly higher CTC count for CC-PCa that reflects the spiking experiments. Furthermore, the weak correlation of the CTC counts between the different functionalized CellCollector systems demonstrated the independence of the CTC counts. The only difference between the CellCollector systems was the surface antibody functionalization for capturing CTCs. The immunocytochemistry characterization of the CTCs was identical and performed by the same operator. These results demonstrate the heterogeneity in the CTC population based on different cell-surface antigens. CC-PCa allowed identification of a hybrid population of CTCs, which may reflect epithelial/mesenchymal phenotypes. EMT is a crucial process of tumor progression [40]. In the case of our analysis, in addition to the EpCAM signal, the isolated CTCs expressed PSMA, PSA and/or PSCA. In other studies, the detection rate of PSMA-positive CTCs ranged between 67% [41] and 59% [42] in advanced PCa patients. In our previous clinical trial, we detected a PSMA signal at the mRNA level in CTCs isolated with CC-EpCAM in only 14.3% of mPCa patients [32]. Nagaya et al. [42] observed that increased PSMA expression in CTCs was associated with a poor treatment response and shorter OS and PSA progression-free survival in castration-resistant PCa patients. In contrast, we observed in cohort 1 no significant differences in OS between the different functionalized CellCollector systems. Nevertheless, interestingly, patients with ≥5 CTCs isolated with CC-EpCAM had an OS of 0.93 years, and patients with ≥5 CTCs isolated with CC-PCa had an OS of 1.5 years in this cohort. Even if advanced PCa patients have more CTCs, these cells can lose the features of epithelial cells and undergo EMT, thereby promoting disease progression [9,43]. However, Keller et al. [44], in 2019, reviewed the biological functions of EpCAM, including the regulation of cell proliferation and cancer stemness, and emphasized the active role of EpCAM in cancer metastasis. Therefore, it is essential for CTC isolation platforms based on biological characteristics to not completely eliminate EpCAM molecules from capturing strategies. This allows the capture of CTCs with mesenchymal and epithelial phenotypes and of various intermediate stages [10].

Markou et al. [31] demonstrated, in a multiplex gene expression profiling of EpCAM-positive CTCs, the detection of stem cell markers (CD133, ALDH1A1 and PSCA) only before surgery or radiotherapy. However, the EMT markers TWIST1, VIM, CDH2 and B2M in isolated CTCs after surgery/radiotherapy in high-risk PCa patients were increased. They concluded that CTCs with an EMT phenotype remain undetected by EpCAM-based isolation methods [31]. CC-PCa, which was developed in our investigation, allowed the capture of such CTCs without a complete exclusion of EpCAM capture.

The Epic Science platform carries out CTC isolation without specific selection, only with red blood cell lysis and deposition of all nucleated cells on 12 glass slides [45]. Scher H. and colleagues quantified 9225 individual CTCs of 179 metastatic CRPC patients isolated with this platform and defined phenotypically distinct cell types [46]. They showed that phenotypic heterogeneity was associated with OS and treatment response. They demonstrated that patients with low heterogeneity in CTC population benefit from treatment with androgen receptor signaling inhibitors, whereas patients with high heterogeneity in CTCs benefit from taxane chemotherapy [46]. These results also confirm the significance and relevance of the CTC heterogeneity evaluation.

In cohort 2, we evaluated different CTC isolation platforms based on physical CTC properties and biological properties based on EpCAM expression. The greatest advantage of antibody-independent platforms is unattached cells. Antibodies or biomolecules for CTC capture may influence downstream analysis [47]. We observed that the CTC detection rates have a broad range independent of the physical or biological CTC properties. The filter system (SC) allowed CTC selection by size and had the highest detection rate in our investigation. Similar results were presented in blood samples of metastatic breast cancer [48] and in lung cancer patients [49] with this filter system. Interestingly, CC-EpCAM reached second place in detection rate and CTC count, which confirms the discussion above of the role of EpCAM-based CTC isolation in advanced PCa patients. The highest CTC count was determined with the SC filter system and MGG staining. The advantage of this system is that CTC selection is based only on the size of the CTCs. Red blood cells (8 µm) pass through the filter. White blood cells (7–12 µm) sometimes fall between pores, but they can be identified on the basis of nuclear morphology and size. The disadvantage of this system is blood clots on the filters, which result in loss of blood samples without any results. A reason for clotting may be a high concentration of CTC clusters (>8 µm), which have a higher metastatic potential through increased cell survival and reduced apoptosis [50]. Furthermore, smaller CTCs (4–8 µm) can run through the filter, which reduces the possibility to analyze the morphological and phenotypical [51] heterogeneity of CTC population.

The lower CTC counts of the Ficoll * systems and the CC-EpCAM system may be the result of the multiple washing and sample transfer steps. The Ficoll * system had the most procedural steps with possible loss of CTCs. However, our research shows the Ficoll * systems had the highest sensitivity and specificity for 24-month survival. On the contrary, the CC-PCa and CC-EpCAM have lower specificity but higher sensitivity of CTC capturing compared to the Ficoll * system. Furthermore, in our previous trial, the EpCAM wire demonstrated in vivo a similar diagnostic accuracy to the FDA-approved CellSearch System [28] which confirmed in vivo application of the CellCollector. Unexpectedly, CTC cultivation was successful only with the Ficoll * system. This isolation method offered nearly untouched CTCs and minimized the shear stress of cells. CTCs were captured from patients who were treated with ADT and had progression of the cancer stage (more than five metastases in bone lymph). The CTC cell line cultivation period was possible for over 3 weeks. The cells secreted PSA at the highest concentration on the 10th day of cultivation. It is possible that the cells changed their PSA expressions under cell culture conditions, and only single cells were positive for PSA signals in immunohistochemistry. CTCs were positive for CK 8, 18 and 19, which confirmed their epithelial state. The PSA level of the patient after 2 months was slightly reduced under ADT, which could be caused by initiation of the intrinsic apoptotic pathway in CTCs. However, we have not analyzed apoptotic markers.

Koch et al. [52] described, in their investigation of a CTC-derived breast cancer cell line, that a high number of CTCs is necessary for the establishment of cell line analysis. We had experiences that a smaller number of CTCs isolated with Ficoll ** was sufficient for culture, although only for a limited time. This suggests the differences in phenotype and high cell plasticity of both cell lines, which may also represent heterogenic stimuli [53].

The detection of CTC in our patient could indicate the dynamic of tumor growth; however, it reflects only the circumstances of one time point. To describe the correlations between the CTC count and clinical behavior of PCa patients, the monitoring must be continued for a longer time. After we compare the CTC isolation methods, we can plan such studies.

Our study has limitations, including the small sample size. The platform results were obtained by manual enumeration of cells by immunocytochemistry or MGG. Visual identification of CTCs was performed with a higher-level trained operator but can still be subjective.

In summary, the best detection rates and highest CTC counts in our investigation were reached with the CC-PCa and SC systems. Nevertheless, we observed the highest sensitivity and specificity for 24-month survival with the Ficoll * and SC platforms with MGG staining. The EpCAM molecule is an essential factor related to OS for CTC isolation based on biological properties in mPCa patients. We observed the highest purity by the ex vivo use of the CellCollector system. Cultivation of CTCs was only possible with Ficoll *. The cost of the platforms and the required skills of the operator are similar. We used standardized conditions for the isolation and characterization of CTCs and short sample transfer. We conclude that the best-suited CTC capture technique is not limited to one characteristic of the cells and must be adapted to the downstream analysis. Our results imply that the clinical importance of the different CTC phenotypes must be elucidated.

## Figures and Tables

**Table 1:** Clinical characteristics of the metastatic prostate cancer patients in cohorts 1 and 2.

	Cohort 1	Cohort 2	*p* Value
Patients, *n* (%)	15 (100)	10 (100)	
Median age (range), years	74 (60–84)	63.5 (49–86)	0.03
Ethnicity	Caucasian	Caucasian	
Gleason score at diagnosis, *n* (%)			0.09
≤7	3 (20)	1 (10)	
>7	12 (80)	9 (90)	
PSA at study visit, median ng/mL (range)	13 (0.02–353)	71.9 (1.7–184)	0.8
Primary therapy, *n* (%)			
Surgery	6 (40)	3 (30)	0.69
Radiation	9 (60)	5 (50)	0.69
Current therapy, *n* (%)			
Androgen deprivation	15 (100)	10 (100)	1
TURP	15 (100)	2 (20)	<0.001
Chemotherapy	10 (83.3)	8 (80)	0.66
Site of metastatic disease, *n* (%)			
Bone	12 (86.7)	10 (100)	0.14
Lymph	5 (41.7)	5 (50)	0.68
Brain	1 (8.3)		
Liver	1 (8.3)		

Abbreviations: PSA = prostate-specific antigen, TURP = transurethral resection of the prostate, CTC = circulating tumor cells.

**Table 2:** Receiver operating characteristic curves for the CTC isolation methods.

CTC Isolation Method	AUC	95% CI	*p* Value
Cohort 1: CC-EpCAM	0.53	0.22–0.84	0.86
Cohort 1: CC-PCa	0.67	0.38–0.95	0.29
Cohort 2: CC-EpCAM	0.55	0.14–0.96	0.82
Cohort 2: SC-MMG	0.73	0.36–1.00	0.30
Cohort 2: Ficoll *	0.79	0.50–1.00	0.17
Cohort 2: Ficoll **	0.67	0.30–1.00	0.44

Abbreviations: CC-EpCAM = CellCollector EpCAM functionalized, CC-PCa = CellCollector PCa functionalized, SC-MMG = SC filter with MGG staining. * MGG staining, ** immunofluorescence staining.

**Table 3:** Characteristics of different CTC isolation methods.

Method	CellCollector EpCAM	CellCollector PCa	ScreenCell	Ficoll *
CTC isolation basis	EpCAM	PSMA, PSA, PSCA, EpCAM	Size: <8 µm cells	Density of Cell + CD45
Used marker				
	CK 8, 18,19, CD45	CK 8, 18, 19, CD45	CK8, 18, 19, CD45	CK 8, 18, 19, CD45
			or MGG	or MGG
Test volume (EDTA blood)	7.5 mL	7.5 mL	3 mL	3 mL
Leukocyte contamination	<10	<10	≥2000	≥500
CTC cultivation effort	n.p.	n.p.	no	yes
Hands-on time for the CTC result per test	≥3 h	≥3 h	≥2 h	≥3 h
Cost per test	±300 €	±350 €	±250 €	±200 €

Abbreviations: EpCAM = epithelial cell adhesion molecule, PSMA = prostate-specific membrane antigen, PSA = prostate-specific antigen, PSCA = prostate-specific stem cell antigen, CTC = circulating tumor cell, CD45 = cluster of differentiation 45, MGG = May-Grunwald Giemsa, n.p.

## Data Availability

The data are not publicly available due to privacy and ethical restrictions.

## References

[1] Lambert A.W., Pattabiraman D.R., Weinberg R.A. (2017). Emerging biological principles of metastasis. Cell.

[2] Tellez-Gabriel M., Heymann M.F., Heymann D. (2019). Circulating Tumor Cells as a Tool for Assessing Tumor Heterogeneity. Theranostics.

[3] Welch D.R., Hurst D.R. (2019). Defining the hallmarks of metastasis. Cancer Res..

[4] Massague J., Obenauf A.C. (2016). Metastatic colonization by circulating tumour cells. Nature.

[5] Kong D., Banerjee S., Ahmad A., Li Y., Wang Z., Sethi S., Sarkar F.H. (2010). Epithelial to mesenchymal transition is mechanistically linked with stem cell signatures in prostate cancer cells. PLoS ONE.

[6] Yang M., Zhang X., Guo L., Liu X., Wu J., Zhu H. (2021). Research progress for the clinical application of circulating tumor cells in prostate cancer diagnosis and treatment. BioMed Res. Int..

[7] Haffner M.C., Zwart W., Roudier M.P., True L.D., Nelson W.G., Epstein J.I., De Marzo A.M., Nelson P.S., Yegnasubramanian S. (2021). Genomic and phenotypic heterogeneity in prostate cancer. Nat. Rev. Urol..

[8] Sartor O., de Bono J.S. (2018). Metastatic prostate cancer. N. Engl. J. Med..

[9] Theil G., Fornara P., Bialek J. (2020). Position of circulating tumor cells in the clinical routine in prostate cancer and breast cancer patients. Cancers.

[10] Keller L., Pantel K. (2019). Unravelling tumour heterogeneity by single-cell profiling of circulating tumour cells. Nat. Rev. Cancer.

[11] Alix-Panabieres C., Pantel K. (2014). Technologies for detection of circulating tumor cells: Facts and vision. Lab Chip.

[12] Cristofanilli M. (2006). Circulating tumor cells, disease progression, and survival in metastatic breast cancer. Semin. Oncol..

[13] De Bono J.S., Scher H.I., Montgomery R.B., Parker C., Miller M.C., Tissing H., Doyle G.V., Terstappen L.W., Pienta K.J., Raghavan D. (2008). Circulating tumor cells predict survival benefit from treatment in metastatic castration-resistant prostate cancer. Clin. Cancer Res..

[14] Cristofanilli M., Budd G.T., Ellis M.J., Stopeck A., Matera J., Miller M.C., Reuben J.M., Doyle G.V., Allard W.J., Terstappen L.W. (2004). Circulating tumor cells, disease progression, and survival in metastatic breast cancer. N. Engl. J. Med..

[15] Cohen S.J., Punt C.J., Iannotti N., Saidman B.H., Sabbath K.D., Gabrail N.Y., Picus J., Morse M., Mitchell E., Miller M.C. (2008). Relationship of circulating tumor cells to tumor response, progression-free survival, and overall survival in patients with metastatic colorectal cancer. J. Clin. Oncol..

[16] Santoni M., Scarpelli M., Mazzucchelli R., Lopez-Beltran A., Cheng L., Cascinu S., Montironi R. (2014). Targeting prostate-specific membrane antigen for personalized therapies in prostate cancer: Morphologic and molecular backgrounds and future promises. J. Biol. Regul. Homeost. Agents.

[17] Heston W.D. (1997). Characterization and glutamyl preferring carboxypeptidase function of prostate specific membrane antigen: A novel folate hydrolase. Urology.

[18] Evans M.J., Smith-Jones P.M., Wongvipat J., Navarro V., Kim S., Bander N.H., Larson S.M., Sawyers C.L. (2011). Noninvasive measurement of androgen receptor signaling with a positron-emitting radiopharmaceutical that targets prostate-specific membrane antigen. Proc. Natl. Acad. Sci. USA.

[19] Chang S.S., O’Keefe D.S., Bacich D.J., Reuter V.E., Heston W.D., Gaudin P.B. (1999). Prostate-specific membrane antigen is produced in tumor-associated neovasculature. Clin. Cancer Res..

[20] Gu Z., Thomas G., Yamashiro J., Shintaku I.P., Dorey F., Raitano A., Witte O.N., Said J.W., Loda M., Reiter R.E. (2000). Prostate stem cell antigen (PSCA) expression increases with high gleason score, advanced stage and bone metastasis in prostate cancer. Oncogene.

[21] Reiter R.E., Sato I., Thomas G., Qian J., Gu Z., Watabe T., Loda M., Jenkins R.B. (2000). Coamplification of prostate stem cell antigen (PSCA) and MYC in locally advanced prostate cancer. Genes Chromosomes Cancer.

[22] Zhigang Z., Wenlv S. (2004). Prostate stem cell antigen (PSCA) expression in human prostate cancer tissues: Implications for prostate carcinogenesis and progression of prostate cancer. Jpn. J. Clin. Oncol..

[23] van der Toom E.E., Axelrod H.D., de la Rosette J.J., de Reijke T.M., Pienta K.J., Valkenburg K.C. (2019). Prostate-specific markers to identify rare prostate cancer cells in liquid biopsies. Nat. Rev. Urol..

[24] Epstein J.I. (1993). PSA and PAP as immunohistochemical markers in prostate cancer. Urol. Clin. N. Am..

[25] Kim J., Coetzee G.A. (2004). Prostate specific antigen gene regulation by androgen receptor. J. Cell. Biochem..

[26] Miyamoto D.T., Lee R.J., Stott S.L., Ting D.T., Wittner B.S., Ulman M., Smas M.E., Lord J.B., Brannigan B.W., Trautwein J. (2012). Androgen receptor signaling in circulating tumor cells as a marker of hormonally responsive prostate cancer. Cancer Discov..

[27] Smith M.R., Cook R., Lee K.A., Nelson J.B. (2011). Disease and host characteristics as predictors of time to first bone metastasis and death in men with progressive castration-resistant nonmetastatic prostate cancer. Cancer.

[28] Theil G., Boehm C., Fischer K., Bialek J., Hoda R., Weber E., Schönburg S., Kawan F., Fornara P. (2021). In vivo isolation of circulating tumor cells in patients with different stages of prostate cancer. Oncol. Lett..

[29] Chen S., Tauber G., Langsenlehner T., Schmölzer L.M., Pötscher M., Riethdorf S., Kuske A., Leitinger G., Kashofer K., Czyż Z.T. (2019). In Vivo Detection of Circulating Tumor Cells in High-Risk Non-Metastatic Prostate Cancer Patients Undergoing Radiotherapy. Cancers.

[30] Saucedo-Zeni N., Mewes S., Niestroj R., Gasiorowski L., Murawa D., Nowaczyk P., Tomasi T., Weber E., Dworacki G., Morgenthaler N.G. (2012). A novel method for the in vivo isolation of circulating tumor cells from peripheral blood of cancer patients using a functionalized and structured medical wire. Int. J. Oncol..

[31] Markou A., Lazaridou M., Paraskevopoulos P., Chen S., Świerczewska M., Budna J., Kuske A., Gorges T.M., Joosse S.A., Kroneis T. (2018). Multiplex Gene Expression Profiling of In Vivo Isolated Circulating Tumor Cells in High-Risk Prostate Cancer Patients. Clin. Chem..

[32] Theil G., Fischer K., Weber E., Medek R., Hoda R., Lücke K., Fornara P. (2016). The Use of a New CellCollector to Isolate Circulating Tumor Cells from the Blood of Patients with Different Stages of Prostate Cancer and Clinical Outcomes—A Proof-of-Concept Study. PLoS ONE.

[33] Allard W.J., Matera J., Miller M.C., Repollet M., Connelly M.C., Rao C., Tibbe A.G., Uhr J.W., Terstappen L.W. (2004). Tumor cells circulate in the peripheral blood of all major carcinomas but not in healthy subjects or patients with nonmalignant diseases. Clin. Cancer Res..

[34] Schoenfeld D.A. (1983). Sample-size formula for the proportional-hazards regression model. Biometrics.

[35] Peduzzi P., Concato J., Feinstein A.R., Holford T.R. (1995). Importance of events per independent variable in proportional hazards regression analysis. II. Accuracy and precision of regression estimates. J. Clin. Epidemiol..

[36] Agashe R., Kurzrock R. (2020). Circulating tumor cells: From the laboratory to the cancer clinic. Cancers.

[37] Ma N., Jeffrey S.S. (2020). Deciphering cancer clues from blood. Science.

[38] Wang D., Wang Z., Tian J., He X., Chowdhury W.H., Zhang X., Li S., Rodriguez R. (2010). Prostate stem cell antigen enhancer and uroplakin II promoter based bladder cancer targeted tissue-specific vector. Urol. Oncol..

[39] Kallergi G., Politaki E., Alkahtani S., Stournaras C., Georgoulias V. (2016). Evaluation of isolation methods for circulating tumor cells (CTCs). Cell. Physiol. Biochem..

[40] Brabletz T., Kalluri R., Nieto M.A., Weinberg R.A. (2018). EMT in cancer. Nat. Rev. Cancer.

[41] Gorges T.M., Riethdorf S., von Ahsen O., Nastały P., Röck K., Boede M., Peine S., Kuske A., Schmid E., Kneip C. (2016). Heterogeneous PSMA expression on circulating tumor cells: A potential basis for stratification and monitoring of PSMA-directed therapies in prostate cancer. Oncotarget.

[42] Nagaya N., Nagata M., Lu Y., Kanayama M., Hou Q., Hotta Z.U., China T., Kitamura K., Matsushita K., Isotani S. (2020). Prostate-specific membrane antigen in circulating tumor cells is a new poor prognostic marker for castration-resistant prostate cancer. PLoS ONE.

[43] Werner S., Stenzl A., Pantel K., Todenhofer T. (2017). Expression of epithelial mesenchymal transition and cancer stem cell markers in circulating tumor cells. Adv. Exp. Med. Biol..

[44] Keller L., Werner S., Pantel K. (2019). Biology and clinical relevance of EpCAM. Cell Stress.

[45] Werner S.L., Graf R.P., Landers M., Valenta D.T., Schroeder M., Greene S.B., Bales N., Dittamore R., Marrinucci D. (2015). Analytical validation and capabilities of the epic CTC platform: Enrichment-free circulating tumour cell detection and characterization. J. Circ. Biomark..

[46] Scher H.I., Graf R.P., Schreiber N.A., McLaughlin B., Jendrisak A., Wang Y., Lee J., Greene S., Krupa R., Lu D. (2017). Phenotypic heterogeneity of circulating tumor cells informs clinical decisions between AR signaling inhibitors and taxanes in metastatic prostate cancer. Cancer Res..

[47] Eslami S.Z., Cortés-Hernández L.E., Cayrefourcq L., Alix-Panabières C. (2020). The different facets of liquid biopsy: A kaleidoscopic view. Cold Spring Harb. Perspect. Med..

[48] Drucker A., Teh E.M., Kostyleva R., Rayson D., Douglas S., Pinto D.M. (2020). Comparative performance of different methods for circulating tumor cell enrichment in metastatic breast cancer patients. PLoS ONE.

[49] Freidin M.B., Tay A., Freydina D.V., Chudasama D., Nicholson A.G., Rice A., Anikin V., Lim E. (2014). An assessment of diagnostic performance of a filter-based antibody-independent peripheral blood circulating tumour cell capture paired with cytomorphologic criteria for the diagnosis of cancer. Lung Cancer.

[50] Aceto N., Bardia A., Miyamoto D.T., Donaldson M.C., Wittner B.S., Spencer J.A., Yu M., Pely A., Engstrom A., Zhu H. (2014). Circulating tumor cell clusters are oligoclonal precursors of breast cancer metastasis. Cell.

[51] Dong L., Zhang Z., Smith K., Kuczler M.D., Reyes D., Amend S.R., Cho Y.K., Xue W., Pienta K.J. (2020). The combination of size-based separation and selection-free technology provides higher circulating tumour cells detection sensitivity than either method alone in patients with metastatic prostate cancer. BJU Int..

[52] Koch C., Kuske A., Joosse S.A., Yigit G., Sflomos G., Thaler S., Smit D.J., Werner S., Borgmann K., Gärtner S. (2020). Characterization of circulating breast cancer cells with tumorigenic and metastatic capacity. EMBO Mol. Med..

[53] Tayoun T., Faugeroux V., Oulhen M., Aberlenc A., Pawlikowska P., Farace F. (2019). CTC-derived models: A window into the seeding capacity of circulating tumor cells (CTCs). Cells.

